# Homeostatic plasticity of axonal excitable sites in Alzheimer's disease

**DOI:** 10.3389/fnins.2023.1277251

**Published:** 2023-10-23

**Authors:** Tania Quintela-López, Jonathan Lezmy

**Affiliations:** Department of Neuroscience, Physiology & Pharmacology, University College London, London, United Kingdom

**Keywords:** axon initial segment (AIS), node of Ranvier, Alzheimer's disease, homeostatic plasticity, amyloid pathology, tau pathology

## Introduction

The brain constantly adapts to received inputs by reshaping its neural circuits. These circuits hold the ability to strengthen or depress their activity in mechanisms underlying learning and memory, but also to keep their activity within a normal homeostatic range. Homeostatic adaptations are achieved by maintaining a relatively stable activity set-point in response to aberrant stimuli, for example by scaling the synaptic strength, stabilizing the ratio of excitatory-inhibitory inputs, and modifying the excitable properties of neurons (Turrigiano, 2011). Homeostatic plasticity in somatodendritic compartments is mediated by structural modifications underlying changes in information transmission: changes in the number or size of excitatory and inhibitory synapses, of AMPA, NMDA and GABA receptors, and of voltage-gated Na_v_ and K_v_ channel-mediated currents (Desai, 2003). In addition, homeostatic adaptations were shown to occur at excitable sites along axons, where action potentials (APs) are generated: the axon initial segment (AIS) and the nodes of Ranvier of myelinated axons. These adaptations allow to adjust the output signals by modulating APs along axons. At the AIS, anchoring proteins and ion channels re-localize, and the number of axo-axonic GABAergic synapses is adjusted to tune neurons' excitability (Grubb and Burrone, 2010; Kuba et al., 2010; Pan-Vazquez et al., 2020). Nodes, which have a similar molecular composition to that of the AIS, also share the ability to modify their length in response to stimuli and may regulate the information flow along myelinated axons in a homeostatic manner (Cullen et al., 2021).

**Figure 1:**
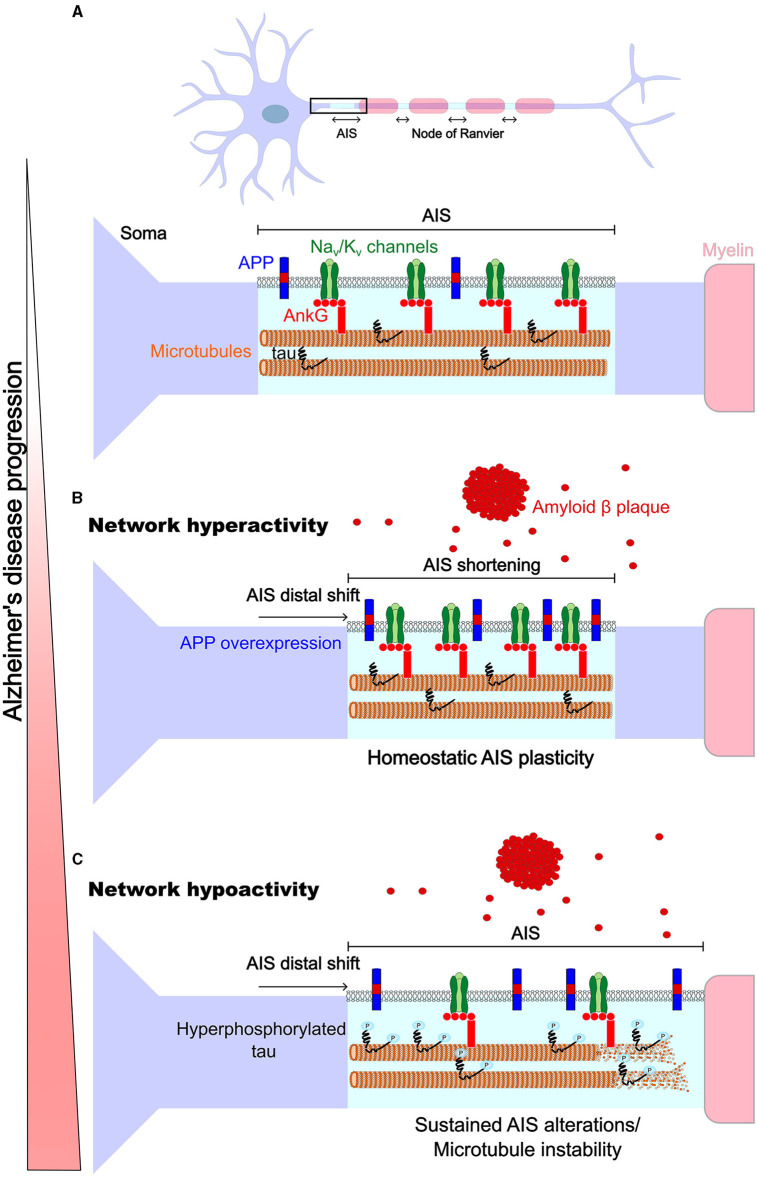
Homeostatic plasticity of axonal excitable sites in Alzheimer's disease. **(A)** *Top*, The AIS and nodes of Ranvier share a similar molecular composition and may be subject to similar homeostatic adaptation mechanisms, but nodes have been less studied (thus nodes are not represented in detail in the diagram). *Bottom*, the AIS, like the nodes, highly expresses voltage-gated Na_v_ and K_v_ channels (green) which are anchored by the structural proteins βIV-spectrin (not shown) and ankyrin G (light red), and ankyrin G binds to microtubules (orange and beige). Endogenous transmembrane protein APP (blue and red) and intracellular tau (black) are expressed at axonal excitable sites. **(B)** Network hyperactivity early in AD is associated with amyloid pathology, where APP is overexpressed, high levels of Aβ are secreted extracellularly and Aβ plaques are formed (red). At this stage, an AIS shift away from the soma and a shortening of the AIS were reported. Thus, modifications of the AIS could be driven by mechanisms of homeostatic plasticity that counter network hyperactivity. **(C)** Network hypoactivity occurs at a later stage and is associated with tau pathology which follows amyloid pathology. Here, a shift of the AIS away from the soma was reported, which could reinforce the neuronal hypoactivity. This may be due to sustained AIS modifications, aberrantly mimicking the homeostatic adaptations triggered by the earlier amyloid pathology, and/or due to AIS structural modifications mediated by tau hyperphosphorylation causing the loss of microtubule stability (which may precede AIS disassembly).

Alzheimer's disease (AD), the commonest cause of dementia, is linked to lapses in memory from its early stages due to synapse loss, followed by a cognitive decline due to ongoing neurodegeneration. It has been proposed that impairments in synaptic homeostatic plasticity mechanisms may underlie early symptoms of the disease (Frere and Slutsky, 2018; Taylor and Jeans, 2021). Interestingly, AIS homeostatic adaptations which regulate neurons' excitability may occur before homeostatic scaling of synaptic activity (Lezmy et al., 2020), raising the question whether axonal homeostatic plasticity mechanisms are involved from the earliest stages of the disease. Here, we will review recent studies showing that axonal excitable sites are targets in AD, focussing on amyloid and tau pathologies (Figure 1). Finally, we will discuss how these disruptions may interfere with axonal homeostatic adaptations and contribute to AD progression.

## Disruption of the AIS structure in amyloid pathology

Early in AD, extracellular amyloid β (Aβ) accumulates as amyloid-beta precursor protein (APP) is cleaved, and aggregates of Aβ molecules form amyloid plaques. Studies suggest that AIS structural alterations may be driven by their interaction with Aβ plaques. In an Aβ-depositing mouse model (APP/TTA), AIS length did not change in the layers 2–4 of the cortex of 6 months old mice when Aβ plaque burden is moderate, but AISs contacting plaques were shorter in 11 months old mice when Aβ plaque burden is severe (Marin et al., 2016). However, a change in AIS length was not observed in cortical pyramidal neurons of one-year old APP/PS1 mice, another Aβ-depositing model (León-Espinosa et al., 2012). Instead, this model showed a reduction in the number of GABAergic axo-axonic synapses forming on the AISs that contact Aβ plaques. Both models (APP/PS1 and APP/TTA) increase Aβ plaque load by overexpressing APP, up to 10 times more in APP/TTA mice (Marin et al., 2016). Aβ plaques accumulate as early as 6–8 weeks of age in APP/TTA mice (Jankowsky et al., 2005), but only from 6 months old in the APP/PS1 mice used (Jankowsky et al., 2004). In the hippocampus of 18 months old APP^NL − F^ mice, a knock-in model expressing a mutant APP (thus avoiding artifacts resulting from APP overexpression) where plaques start accumulating at 6 months old (Saito et al., 2014), GABAergic synapses at the AIS were enlarged, while the total number of synapses at the AIS was unchanged (changes in AIS length were not reported here; Sos et al., 2020). Thus, although the underlying mechanism is unclear, Aβ plaque load may promote AIS structural alterations as AD progresses, by modifying the AIS length and shaping the inhibitory inputs that the AIS receives.

Alterations in the AIS structure may be mediated by APP overexpression or soluble Aβ rather than, or in addition to, Aβ plaque toxicity. In cortical and hippocampal APP/PS1 primary neurons (where APP and soluble Aβ levels, but not plaques, is expected to be high), AISs were shorter than in wild-type (WT) mice and were not responsive to homeostatic changes in AIS length usually triggered by increasing (with bicuculline) or decreasing (with TTX) neuronal activity (Martinsson et al., 2022). Alternatively, applying soluble Aβ onto cultured hippocampal neurons impaired microtubule plus-end-binding protein 3 (EB3) stability at the AIS, thereby disturbing microtubule integrity and lengthening the AIS, and was accompanied by a depolarising shift in the AP voltage threshold (Tsushima et al., 2015). This effect was mediated by Aβ inhibition of histone deacetylase 6 (HDAC6), as applying tubacin (a HDAC6 inhibitor) increased EB3 dynamics at the AIS and lengthened the AIS, similar to Aβ application. A recent study suggests that APP overexpression induced by glutamate excitotoxicity, or by overexpression via plasmid transfection carrying the APP_Swe_ mutation or using transgenic (R1.40 model) primary neurons, drives the modifications in AIS structure (Ma et al., 2023). Elevated concentration of APP, which contacts the AIS scaffolding proteins ankyrin G and βIV-spectrin, promoted an AIS shift away from the soma and a shortening of the AIS. In this study, AIS structure was not altered by the interaction with soluble Aβ nor with plaques. Overall, divergent evidence indicates that AIS structural modification may be promoted by either APP, soluble Aβ or Aβ plaques, depending on the mouse model of AD in use, on the age of the mice (and thus the stage of amyloid pathology) and/or on the experimental procedures chosen. We discuss below how some of these findings may be linked to AIS homeostatic adaptation mechanisms.

## Disruption of the AIS structure in tau pathology

Aggregation of tau protein inside neurons occurs after the onset of amyloid pathology in AD (Harris et al., 2020), as tau becomes hyperphosphorylated and dissociates from the microtubule. Hyperphosphorylated tau destabilizes axonal microtubule, and promotes an AIS shift away from the soma and a reduction in the excitability of cultured hippocampal neurons. This AIS distal shift could be prevented by the microtubule stabilizer taxol (Hatch et al., 2017). Furthermore, in CA1 pyramidal neurons of rTg4510 mice (where mutant tau is overexpressed, and thus have more hyperphosphorylated tau), the AIS was shifted away from the soma and the neuronal excitability was reduced, months before the onset of neurodegeneration in this tauopathy model (Hatch et al., 2017). In contrast, a recent study shows that, in brains from AD patients *post-mortem*, AISs of cortical pyramidal neurons with hyperphosphorylated tau (immunolabeled with AT8) were commonly found to be closer to the soma and longer (Antón-Fernández et al., 2022). Thus, as for amyloid pathology, disease stage may be crucial in defining the influence of tau on the AIS structure. To assess tau effects on AIS alterations, Chang et al. (2021) used a mouse model where tau is ablated (MAPT knockout). In the layer 5 of the somatosensory cortex, they observed a shortening of the AIS in excitatory pyramidal cells, and a lengthening of the AIS together with a shift closer to the soma in inhibitory parvalbumin (PV+) neurons. Furthermore, in tau knockout mice, the AIS of PV+ interneurons, but not of excitatory neurons, was irresponsive to chronic depolarisation that usually triggers an AIS shift away from the soma in wild-type mice. Thus, endogenous tau, which interacts with EB3 to stabilize ankyrin G in the AIS, may be involved in AIS homeostatic adaptation mechanisms in a cell type-dependent manner.

## Disruption of the node of Ranvier structure in AD

Homeostatic adaptations at nodes of Ranvier have been much less studied than their counterparts at the AIS. Since nodes and AISs have a similar molecular composition, where Na_v_ and K_v_7 channels are highly expressed and anchored by ankyrin G and βIV-spectrin, it is reasonable to assume that at least some of the mechanisms involved in Aβ and tau pathologies discussed above apply to nodes, although experimental studies verifying this assumption will be needed in the future. Interestingly, APP was shown to be highly expressed in nodes of the optic nerve, cerebellum and spinal cord (but not in nodes of the peripheral nervous system). APP may be involved in regulating node length, as APP KO mice had longer nodes than wild-type mice (Xu et al., 2014). Furthermore, the conduction speed of spinal cord axons in APP KO mice was reduced, as well as the Na^+^ current density of CA1 pyramidal neurons. Na_v_1.6-mediated currents were elevated in HEK293 cell lines overexpressing APP (Li et al., 2016), as APP phosphorylation increased Na_v_1.6 surface expression (Liu et al., 2015). In addition, the C99 fragment of APP, which accumulates intracellularly in AD, binds to Kv7.2/3 channels at nodes and inhibits their activity (Manville and Abbott, 2021). Thus, high APP levels may not only induce alterations in the structure of the AIS and nodes but also directly affect the activity of ion channels shaping APs at axonal excitable sites. In this article, we do not discuss impairments in glia-axon interaction nor in myelination in AD (reviewed recently in Nasrabady et al., 2018; Hirschfeld et al., 2022; Uddin and Lim, 2022; Maitre et al., 2023), but it is important to mention that changes in glial responses and in myelin pattern may also modify the structure of the AIS and nodes. However, it is unclear whether glia-axon and myelin-mediated alterations of excitable sites would occur as a result of a disbalance in homeostatic plasticity mechanisms applying on the myelin and glia, or as a consequence of myelin damage and glia aberrant activity linked to AD.

## Link between homeostatic axonal plasticity and Alzheimer's disease

Evidence described above suggests that, at least in some instances, alteration in the structure of axonal excitable sites may reflect disruption of mechanisms underlying homeostatic axonal plasticity. To speculate on this, we aligned the occurrence of the AIS alterations described above with the known changes in neuronal activity at different stages of AD (Figure 1). Although AD is a complex disease that alter many cellular mechanisms with divergent effects on neural circuits, initial Aβ rise can be broadly associated with network hyperactivity, while the later accumulation of tau can be associated with network hypoactivity (Harris et al., 2020). The initial hyperactivity may explain the finding of short AISs when Aβ plaque load is severe (Marin et al., 2016), when soluble Aβ accumulates (Martinsson et al., 2022) and when APP is overexpressed (Martinsson et al., 2022; Ma et al., 2023). An AIS shortening is expected to reduce neuronal excitability to compensate for prolonged hyperactivity in healthy neurons (although it is not always the case; see Adachi et al., 2015). The AIS distal shift found in association with APP overexpression is also expected to lower the neuronal excitability (Ma et al., 2023). In addition, larger GABAergic synapses at the AIS in the APP^NL − F^ model reinforce the suggestion that the AIS reshapes to counter network hyperexcitability (Sos et al., 2020). However, less GABAergic synapses were found in the APP/PS1 (León-Espinosa et al., 2012), which would presumably increase neuronal excitability, opposing a homeostatic effect, although regulation of synapse number at the AIS crucially depends on whether the synapses are depolarising or hyperpolarising (Pan-Vazquez et al., 2020). Prolonged application of Aβ onto neurons also reduced neuronal excitability (Tsushima et al., 2015), but this could also reflect a loss of integrity of the AIS, as the AIS lengthens because microtubule disassembles when tau acetylates.

Generally, pathogenic tau accumulation would result in a shift of the AIS away from the soma in excitatory neurons (Hatch et al., 2017; Chang et al., 2021), which corresponds with an expected decrease in neuronal excitability (Adachi et al., 2015) that should strengthen, rather than compensate for the tau-linked hypoactivity. Tau-mediated effects at the AIS may be mostly driven by microtubule disassembly via tau phosphorylation or acetylation, as part of neurodegeneration processes. Remaining functional axons may attempt to induce homeostatic mechanisms, as the remaining AISs were usually closer to the soma in brains of AD patients *post-mortem* (Antón-Fernández et al., 2022). Although AIS disassembly may explain hyperphosphorylated tau-mediated effects, a possible role for tau in mediating homeostatic changes should not be neglected. Tau mutation (tau^V337M^) or tau knock-out prevented activity-dependent homeostatic AIS plasticity, in mechanisms involving the alteration of microtubule dynamics (Lezmy et al., 2017; Sohn et al., 2019; Chang et al., 2021). From this perspective, endogenous tau-linked homeostatic adaptations could in fact be triggered by the initial Aβ-mediated hyperactivity in AD but, when sustained, could become detrimental in the hypoactive phase linked to pathogenic tau. Finally, disruptions of homeostatic adaptations altering the expression of axonal voltage-gated channels should also be taken into consideration as, although experimental data are limited, changes in their distribution is expected to regulate axonal excitability and conduction speed in health and disease.

## Conclusions

Although the knowledge in the field is still sparse, recent literature suggests that homeostatic plasticity of axonal excitable sites may play a crucial role in the pathophysiology of AD. Axonal homeostatic adaptations that are triggered at the onset of the disease may be beneficial to counter network hyperexcitability. However, sustained or aberrant activation of such mechanisms may become detrimental when AD switches to hypoactivation, and they may then contribute to memory loss and cognitive decline.

## Author contributions

TQ-L: Writing—review and editing. JL: Writing—review and editing, Conceptualization, Writing—original draft.
